# Pain Accelerates, Swelling and Sensory Disturbances Delay Diagnosis in Mesenchymal Tumors

**DOI:** 10.3390/cancers17030510

**Published:** 2025-02-03

**Authors:** Maria Elyes, Philip Heesen, Georg Schelling, Beata Bode-Lesniewska, Gabriela Studer, Bruno Fuchs, on behalf of the Swiss Sarcoma Network

**Affiliations:** 1 Faculty of Health Sciences & Medicine, University Lucerne, Frohburgstrasse 3, 6002 Luzern, Switzerland; maria.elyes@uzh.ch (M.E.);; 2 Medical Faculty, University of Zurich, 8032 Zurich, Switzerland; 3 Sarcoma Service, Department of Orthopedics and Trauma, Sarcoma Center, LUKS University Hospital, 6000 Luzern, Switzerland; 4 Sarcoma Service, Klinik für Orthopädie und Traumatologie, Sarcoma Center, Kantonsspital Winterthur, 8400 Winterthur, Switzerland

**Keywords:** mesenchymal tumors, sarcomas, diagnostic delays, pain, swelling, sensory disturbances, diagnostic intervals, early diagnosis, patient awareness, healthcare provider education

## Abstract

Background/Objectives: The early diagnosis of mesenchymal tumors, including bone and soft tissue sarcoma and their benign counterparts, is crucial for improving patient outcomes. This study aims to analyze how different symptoms influence diagnostic timelines and identify factors contributing to delays in the diagnostic pathway. Methods: We conducted an analysis of 712 patients with mesenchymal tumors from the Swiss Sarcoma Network between January 2018 and December 2021. Symptoms were analyzed both individually, like pain and swelling, and by categories such as general, neurological and integumentary symptoms. Intervals of the total interval (TI) in the diagnostic pathway—including the patient interval (PI), primary care interval (PCI), secondary care interval (SCI) and tertiary care interval (TCI)—were assessed. Linear regression models estimated the impact of specific symptoms on these intervals. Results: Pain significantly reduced the PI and TI in patients with bone sarcomas (PI *β* = −1787.5; *p* = 0.001) and soft tissue sarcomas (TI *β* = −442.1; *p* = 0.04), prompting earlier medical consultation. In contrast, symptoms like growing swelling and sensory disturbances were associated with prolonged diagnostic intervals. Growing swelling lengthened the PI in benign bone tumors (*β* = 382.98; *p* = 0.04) and extended the TI in bone sarcomas (*β* = 1637.4; *p* = 0.02). Sensory disturbances delayed the PI and TI in superficial soft tissue sarcomas (PI *β* = 1705; *p* = 0.02; TI *β* = 2037.6; *p* = 0.03). Integumentary symptoms also led to significant delays in benign superficial soft tissue tumors. Conclusions: Pain accelerates the diagnostic process in mesenchymal tumors by prompting earlier consultation, while swelling and sensory disturbances contribute to delays across various diagnostic intervals. These findings highlight the need for increased awareness among patients and healthcare providers regarding the potential seriousness of these symptoms. Educational initiatives and optimized referral protocols are essential to reducing diagnostic delays and improving patient outcomes.

## Simple Summary

Sarcomas are rare tumors that can develop in bones or soft tissues. Recognizing them early is important because delays in diagnosis can lead to worse outcomes for patients. In our study, we looked at how different symptoms affect the time it takes for patients to be diagnosed with these tumors. We found that when patients experience pain, they tend to see a doctor sooner, leading to quicker diagnoses. However, symptoms like swelling or numbness often cause delays because patients or doctors may not realize they could be signs of a serious condition. Understanding which symptoms lead to delays can help improve education for both patients and healthcare providers, leading to earlier detection and better outcomes for people with sarcomas and similar tumors.

## 1. Introduction

Sarcomas are rare mesenchymal tumors with significant heterogeneity, encompassing both bone and soft tissue neoplasms that can range from benign to malignant and from superficial to deep-seated lesions. This diversity in histological subtypes—over 80 have been identified [1]—and anatomical presentations poses substantial diagnostic challenges. These challenges often result in delayed treatment initiation, which can lead to poorer patient outcomes—a common issue with rare cancers [2]. Early diagnosis is crucial for improving survival rates and enhancing the effectiveness of therapeutic interventions in sarcoma patients [3,4,5]. Therefore, understanding the diagnostic pathway and how various symptoms influence it is vital for improving patient care and outcomes. The non-specific nature of symptoms associated with mesenchymal tumors, such as pain, swelling and sensory disturbances, contributes significantly to diagnostic delays. Patients may not recognize the severity of these symptoms, leading to postponement in seeking medical attention [6]. Additionally, primary care physicians may misinterpret these symptoms due to the rarity and variable clinical presentations, further prolonging the time to accurate diagnosis [7,8,9]. These delays are particularly pronounced in the patient interval (from symptom onset to first medical consultation) and the secondary care interval (from specialist referral to referral to a sarcoma hub), which have been identified as major bottlenecks in the diagnostic pathway [10]. Despite existing research on diagnostic intervals, critical gaps remain unaddressed. Most studies have focused primarily on malignant tumors and common symptoms like pain and lumps, without providing a comprehensive overview of all symptom categories [6]. Benign mesenchymal tumors, as well as the differences between deep and superficial tumors, have been largely understudied. This lack of comprehensive symptom analysis limits the understanding of how various presentations impact the diagnostic process. Furthermore, while the centralization of care has been proposed to improve outcomes—studies have highlighted diagnostic discrepancies when non-specialist centers are involved [11,12]—there is a need for detailed analysis on how specific symptoms affect diagnostic timeliness across different healthcare levels. This study aims to address these gaps by examining how both benign and malignant mesenchymal tumors, as well as deep and superficial tumors, differ in symptom presentation and how these differences impact the diagnostic pathway. By exploring the symptoms, consulted physicians and diagnostic examinations of patients with mesenchymal tumors, we seek to assess their impact on the length of various diagnostic intervals: the patient interval, primary care interval, secondary care interval and tertiary care interval. This comprehensive approach will provide a more detailed understanding of the diagnostic process for sarcoma patients. Early diagnosis, as shown in studies on both general cancers [13] and sarcomas [10,14], significantly improves patient outcomes. Our findings could contribute to earlier detection strategies and more personalized approaches to management, ultimately improving patient outcomes in clinical practice.

## 2. Materials and Methods

### 2.1. Study Design

This study analyzed a prospectively collected dataset from the real-world-time data warehouse (RWTD/E-W) ShapeHub^®^ (PH and BF, Zurich, Switzerland). The dataset comprises patients diagnosed with mesenchymal tumors, including soft tissue sarcomas (STS), bone sarcomas (BS), benign soft tissue tumors (BSTT) and benign bone tumors (BBT). The data were collected from sarcoma centers (MDT/SB-SSN) and their associated network, which includes seven secondary and tertiary care medical institutions forming the Swiss Sarcoma Network (SSN) in Switzerland. The study period spanned from 1 January 2018 to 31 December 2021.

### 2.2. Study Objective

The primary objective of this study was to analyze the symptoms of patients with mesenchymal tumors and to assess their impact on the length of various intervals of the diagnostic pathway: the patient interval (PI), primary care interval (PCI), secondary care interval (SCI) and tertiary care interval (TCI). This analysis was conducted across subgroups of STS, BS, BSTT and BBT. The study aimed to identify factors contributing to delays or efficiencies in each interval, with the long-term goal of optimizing diagnostic pathways to improve the timely detection and treatment of sarcomas and other mesenchymal tumors.

### 2.3. Selection Criteria

**Figure 1:**
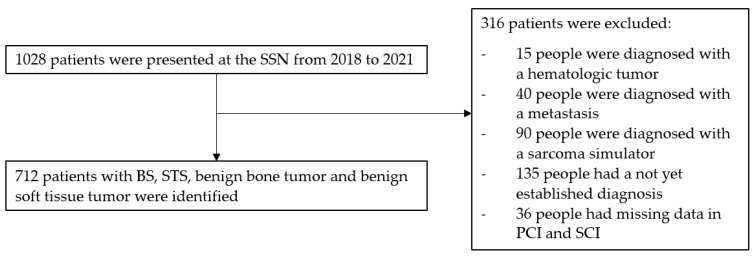
Flow chart of the patient inclusion progress.

All consecutive patients presented at the weekly MDT/SB-SSN meetings with a diagnosis of STS, BS, BSTT or BBT between 1 January 2018 and 31 December 2021 were considered for inclusion. Diagnoses were established based on the World Health Organization (WHO) classification and categorized as benign or malignant, with intermediate tumors classified as sarcomas (see Figure 1).

Inclusion Criteria:

- Patients diagnosed with STS, BS, BSTT or BBT within the study period.
- Availability of complete medical records regarding documented symptom presentation and diagnostic timelines.
- Exclusion Criteria:
- Patients with incomplete medical records where key dates (e.g., first physician visit) could not be determined.
- Patients who received prior treatment for mesenchymal tumors before presentation to the SSN.
- Secondary mesenchymal tumors resulting from metastasis.

In the Swiss healthcare system, visiting a primary care physician is not mandatory before consulting a specialist. Therefore, patients with incomplete data regarding the PCI were still included, as it was not possible to differentiate between the absence of physician-directed care and missing documentation of a physician visit during the PCI. The same approach was applied to missing data in the SCI. If medical records explicitly indicated that no primary or secondary care physician was involved—such as incidental findings during other examinations or explicit statements in referral letters—these patients were categorized under “None” in the relevant intervals.

### 2.4. Data Collection

Data were extracted from a real-world-time data warehouse (RWTD/E) managed by Adjumed (Zurich, Switzerland), which aggregates demographic and treatment-specific information from patients across seven Swiss medical institutions. Initially, 1028 patients were identified. After applying the inclusion and exclusion criteria, 712 patients with complete medical records were included in the final analysis. The collected data encompassed the following:

- Demographic Information: Age, sex.
- Tumor Characteristics: WHO diagnosis, histological type, tumor grade (for malignant tumors), size, anatomical location (head and neck, superficial trunk, deep trunk, limb girdle, extremities and viscera) and depth (superficial or deep).
- Symptom presentation: Types of symptoms reported, classified into specific categories.
- Diagnostic Timelines:
  o Date of Symptom Onset: First symptom attributed to the mesenchymal tumor.
  o First Physician Visit: Date of initial medical consultation.
  o Referral Dates: Dates of referral from primary to secondary care and from secondary care to the sarcoma center/tertiary care.
  o Diagnosis Date: Date of histologically confirmed diagnosis.

### 2.5. Definition of the Intervals

- Patient Interval (PI): Time from symptom onset to the first medical consultation.
- Primary Care Interval (PCI): Time from the first medical consultation to referral to secondary care.
- Secondary Care Interval (SCI): Time from secondary care consultation to referral to the sarcoma center.
- Tertiary Care Interval (TCI): Time from sarcoma center consultation to histological diagnosis.
- Diagnostic Interval (DI): Sum of PCI, SCI and TCI, representing the time from the patient’s first medical consultation to the histological diagnosis.
- Total Interval (TI): Sum of PI, PCI, SCI and TCI, representing the total time from symptom onset to the histological diagnosis.

### 2.6. Classification of the Symptoms

Symptoms were categorized based on relevant organ system or clinical domains to provide a structured and clinically meaningful framework:

- Circulatory: Symptoms related to the circulatory system (e.g., edema, cold foot, increased resting heart rate).
- Gastrointestinal: Symptoms related to the digestive tract (e.g., constipation, diarrhea, emesis, loss of appetite, abdominal distension).
- General Symptoms: Non-specific symptoms affecting overall health (e.g., pain, swelling (local increase in volume), fever, fatigue, night sweats, weight loss).
- Integumentary: Symptoms affecting the skin and related tissues (e.g., skin lesions, exophytic tumors, poorly healing wounds, discoloration, ulceration).
- Musculoskeletal: Symptoms related to muscles and bones (e.g., cramps, loss of strength, fasciculations, loss of function).
- Neurological: Symptoms related to the nervous system and sensory functions (e.g., headache, tinnitus, sensory disturbances).
- Respiratory: Symptoms related to breathing and the respiratory tract (e.g., dyspnea, coughing, voice changes).
- Urogenital: Symptoms affecting the urinary and reproductive systems (e.g., acute renal failure, hematuria, dysuria, postmenopausal bleeding).
- Incidental Findings: Unexpected findings detected during routine exams or follow-ups (e.g., incidental findings during cancer control or preoperative examinations), often retrospectively accompanied by symptoms like swelling or pain.
- Not Available (NA): Cases where symptom data were missing or unavailable.

Each symptom category was coded for statistical analysis. Patients presenting with multiple symptoms had each symptom recorded and analyzed accordingly.

### 2.7. Tumor Grading and Classification

The tumor grade was assigned based on the WHO classification system, applicable only to malignant tumors. Intermediate-grade tumors were classified as malignant for the purposes of this study. The tumor size was measured as the largest diameter recorded in imaging studies or surgical reports. The tumor location was categorized as appendicular (limbs) or axial (head, neck, trunk), and the depth was classified as superficial or deep based 175 on anatomical landmarks.

### 2.8. Statiscial Analysis

Continuous variables are presented as medians with interquartile ranges (IQR; 1st quartile, 3rd quartile), while categorical variables are reported as numbers and percentages. According to the study inclusion criteria, a complete case analysis was conducted. Previously analyzed interval lengths, including the patient, primary care, secondary care and tertiary care interval as well as the diagnostic and total interval, serve as baseline data in this study [10]. These intervals are examined here to assess the impact of symptom presentation on diagnostic timelines.

Differences between categorical variables were analyzed using Fisher’s exact test. The association between specific symptoms and intervals of the diagnostic pathway was assessed using linear regression models. Beta coefficients (*β*) with 95% confidence intervals (CI) were calculated to estimate the effect size of symptoms on the PI, PCI, SCI, TCI and TI. A positive *β* indicated a lengthening of the interval, whereas a negative *β* indicated a shortening. Statistical significance was determined as a *p*-value of less than 0.05.

A priori-defined subgroup analyses were performed based on tumor type (malignant vs. benign), location (deep vs. superficial) and other relevant characteristics. All statistical analyses were conducted using R software (version 4.3.2).

### 2.9. Ethical Considerations

The study was approved by the local ethics committee (IRB approval number: BASEC—Nr. 2019-01107/NCT04300257), adhering to the guidelines of the Declaration of Helsinki. Given the retrospective nature of the study and the use of anonymized data, the requirement for informed consent was waived.

## 3. Results

### 3.1. Patient and Tumor Characteristics

#### 3.1.1. Demographic Data

##### Age Distribution

In our cohort of 712 patients with mesenchymal tumors, the median age varied significantly across different tumor groups. Patients with BS had a median age of 44.0 years (interquartile range [IQR]: 19.0–65.0 years), indicating a younger population compared to other groups. STS patients exhibited a higher median age of 60.0 years (IQR: 46.0–72.0 years). BBT patients had a median age similar to BS at 34.0 years (IQR: 23.0–45.0 years), while BSTT presented in an older population with a median age of 55.0 years (IQR: 44.0–63.0 years). Notably, deep-seated tumors, both malignant and benign, tend to occur in older patients compared to superficial tumors (see Table 1). 

##### Gender Distribution

The gender distribution also showed variation among tumor types. Females constituted 36.6% of the BS group, 50.0% of the STS group, 49.2% of the BBT group and 51.6% of the BSTT group. There was a slightly higher prevalence of certain tumors in one gender; for instance, STS had a marginally higher occurrence in males compared to females (see Table 1).

#### 3.1.2. Tumor Characteristics

##### Tumor Grade (Applicable to Malignant Tumors Only)

Among the malignant tumors, tumor grading revealed that a significant proportion were high-grade neoplasms. In BS patients, 31.7% had grade G3 tumors, 6.1% had G2, and 8.5% had G1. Similarly, in the STS group, 28.1% were grade G3, 13.8% were G2 and 18.8% were G1 tumors. High-grade tumors were more prevalent in deep-seated sarcomas compared to superficial ones (see Table 1).

##### Tumor Location

BS were more frequently localized in the extremities (40.2%) and limb girdle (39.0%) compared to STS (30.3% and 22.8%, respectively). Similarly, benign tumors in the extremities were more common, with BBT (52.5%) showing a higher prevalence than BSTT (41.8%). However, BSTT (34.3%) and BBT (29.5%) were found comparably often in the limb girdle. Deep STS were predominantly located in the deep trunk (39.2%), whereas superficial STS were more frequently found in the superficial trunk (36.7%) and accounted for the highest proportion of tumors in the head and neck region (16.7%). Benign deep soft tissue tumors were less often localized in the deep trunk (19.8%) and superficial soft tissue tumors were less often localized in the superficial trunk (14.6%) compared to their malignant counterparts. Tumors in the viscera were observed only in the deep STS group (3.4%) (see Table 1).

##### Tumor Size

The median tumor size also differed among the groups. BS had a median size of 60.0 mm (IQR: 39.5–85.0 mm), while STS tumors were larger, with a median size of 70.0 mm (IQR: 32.0–124.0 mm). Benign tumors generally presented with smaller sizes; BBT had a median size of 31.5 mm (IQR: 11.5–50.5 mm), and BSTT had a median size of 60.0 mm (IQR: 38.3–97.3 mm). Notably, deep tumors were larger on average than superficial ones (see Table 1).

### 3.2. Symptom Prevalence and Impact on the Diagnostic Interval

**Figure 2:**
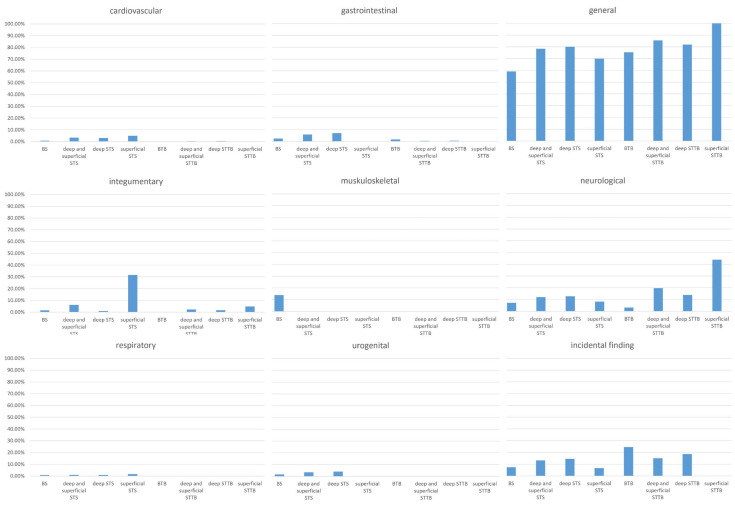
Distribution of Symptom Prevalence (%) Across Different Tumor Groups Categorized by Symptom Class: Circulatory, Gastrointestinal, General, Integumentary, Musculoskeletal, Neurological, Respiratory, Urogenital and Incidental Findings. For a detailed tabular representation and numerical data, please refer to Table A2 in Appendix A.

Analyzing symptom presentations across different tumor groups revealed significant variations in both prevalence (see Figure 2 and Table 2 and Table 3) and their impact on diagnostic intervals (see Table A1). The median lengths of diagnostic pathway intervals are in Table A2 in Appendix A, serving as baseline data for symptom impact analysis.

#### 3.2.1. Circulatory Symptoms

Circulatory symptoms were more frequent in patients with STS, with bleeding reported in 5.0% of these cases, the highest among circulatory symptoms. These symptoms were almost absent in benign tumor groups. Notably, in patients with superficial STS, the presence of circulatory symptoms significantly lengthened the patient interval (PI) (*β* = 2324.5; *p* = 0.006) and the total interval (TI) (*β* = 2804.1; *p* = 0.01). This suggests that patients experiencing bleeding may delay seeking medical attention, possibly due to not associating this symptom with malignancy. Such delays highlight the need for patient education on the significance of bleeding as a potential indicator of serious underlying conditions (see Table 2 and Table 3).

#### 3.2.2. Gastrointestinal Symptoms

Gastrointestinal symptoms were more common in deep malignant tumors, particularly deep STS, where constipation was reported by 2.0% of patients. These symptoms were almost absent in benign tumors. Gastrointestinal symptoms did not have a statistically significant impact on any interval of the diagnostic pathway (PI, diagnostic interval (DI) or TI), indicating that their infrequency and nonspecific nature may not significantly influence patient behavior or diagnostic processes (see Table 2 and Table 3).

#### 3.2.3. General Symptoms

General symptoms, including pain and swelling, were prevalent across several tumor types. Pain was reported by 79.3% of patients with bone sarcomas (BS), 44.3% with deep STS and 39.5% with benign superficial soft tissue tumors (BSTT). Swelling was observed in 75.0% of patients with superficial STS and was also highly prevalent in deep and superficial BSTT. Growing swelling was reported by 35.0% of patients with superficial STS and 37.6% with BSTT. In BS patients, pain significantly shortened the PI (*β* = 1787.5; *p <* 0.001) and the TI (*β* = 2556.8; *p <* 0.001), underscoring its role as a critical symptom prompting earlier medical consultation and facilitating timely diagnosis and treatment. Conversely, swelling did not significantly impact the PI but significantly lengthened the primary care interval (PCI) (*β* = 108.1; *p* = 0.04) and secondary care interval (SCI) (*β* = 134.6; *p* = 0.03) in STS patients. This indicates potential delays in referral and diagnosis due to underrecognition of the symptom’s severity by primary and secondary care providers. Growing swelling significantly lengthened the PI in patients with benign tumors of bone (BBT) (*β* = 383.0; *p* = 0.04) and prolonged the PCI (*β* = 433.2; *p* = 0.007) and SCI (*β* = 433.7; *p* = 0.006) in BS patients, resulting in an extended TI (*β* = 1637.4; *p* = 0.02). These findings suggest that growing swelling may be perceived as less urgent, leading to delays in both patient presentation and progression through the healthcare system. Emphasizing the importance of the timely evaluation of enlarging masses is crucial to prevent diagnostic delays (see Table 2 and Table 3).

#### 3.2.4. Integumentary Symptoms

Integumentary symptoms, such as discoloration and ulcerating swelling, were more common in superficial STS, with discoloration reported in 11.7% and ulceration in 6.7% of patients. Discoloration was also reported in 2.4% of patients with benign superficial BSTT.

In patients with BSTT, integumentary symptoms significantly lengthened the PI (*β* = 2447.1; *p* = 0.002) and the TI (*β* = 2666.1; *p* = 0.009), with even longer delays observed in benign superficial STT (PI *β* = 6596.1; *p <* 0.001; TI *β* = 6216.6; *p <* 0.001). These substantial delays may be due to patients perceiving skin changes as benign or cosmetic issues, underscoring the necessity for public education on the potential seriousness of integumentary symptoms and encouraging prompt medical evaluation (see Table 2 and Table 3).

#### 3.2.5. Musculoskeletal Symptoms

Musculoskeletal symptoms like loss of function were primarily observed in BS (15.9%) and BBT (19.7%) and were rare in other tumor types. While these symptoms showed a tendency to shorten the PI in BS and BBT patients, they significantly prolonged the PCI in benign deep BSTT (*β* = 165.0; *p* = 0.02) and the tertiary care interval (TCI) in BS patients (*β* = 19.1; *p* = 0.02). This suggests the potential misinterpretation or underestimation of musculoskeletal symptoms by healthcare providers, highlighting the need for improved referral pathways and clinician education to reduce delays in diagnosis and treatment initiation (see Table 2 and Table 3).

#### 3.2.6. Neurological Symptoms

Neurological symptoms, particularly sensory disturbances, were most common in benign superficial BSTT (43.9%) and deep STS (11.8%). In superficial STS, neurological symptoms significantly lengthened the PI (*β* = 1705.0; *p* = 0.01) and the TI (*β* = 2037.6; *p* = 0.03), indicating delays in seeking medical attention.

Additionally, sensory disturbances significantly prolonged the PCI and SCI in BS (PCI *β* = 549.5; *p* = 0.002; SCI *β* = 550.1; *p* = 0.002) and benign BSTT (PCI *β* = 153.7; *p* = 0.01; SCI *β* = 134.3; *p* = 0.03). These findings suggest that neurological symptoms may be misattributed to less serious conditions, such as neuropathies or musculoskeletal issues, leading to delays in the diagnostic process. Enhancing awareness among patients and clinicians regarding the potential significance of neurological symptoms in the context of mesenchymal tumors is essential (see Table 2 and Table 3).

#### 3.2.7. Respiratory Symptoms

Respiratory symptoms were rare across all tumor types, with a slight peak in superficial STS, where 1.7% of patients reported dyspnea. Due to their low prevalence, respiratory symptoms did not have a significant impact on intervals of the diagnostic pathway (see Table 2 and Table 3).

#### 3.2.8. Urogenital Symptoms

Urogenital symptoms were slightly more frequent in BS and deep STS, with hematuria reported in 1.2% and 1.0% of patients, respectively. These symptoms did not significantly affect the diagnostic process, likely due to their rarity and nonspecific presentation (see Table 2 and Table 3).

#### 3.2.9. Incidental Findings

Incidental findings were more common in benign tumors, particularly in BBT (24.6%) and benign deep BSTT (18.6%). Such findings emphasize the importance of comprehensive evaluations and imaging studies, even when patients are asymptomatic. They present an opportunity for early detection and management, potentially preventing complications or disease progression (see Table 2).

## 4. Discussion

This study addresses a critical gap in the literature by comprehensively analyzing how various symptoms and symptom classes influence the intervals of the diagnostic pathway in patients with mesenchymal tumors, specifically focusing on bone sarcomas (BS), soft tissue sarcomas (STS) and their benign counterparts. Our findings reveal that pain emerges as a pivotal symptom that significantly reduces the patient interval (PI) and overall total interval in BS and STS patients by prompting earlier medical consultation. Conversely, symptoms like growing swellings, sensory disturbances and integumentary symptoms were associated with diagnostic delays, extending intervals such as the PI, primary care interval (PCI), secondary care interval (SCI) and tertiary care interval (TCI) in both malignant and benign tumors. Existing literature primarily emphasizes pain and lumps as the predominant symptoms reported by sarcoma patients [15,16,17,18]. However, their influence on intervals of the diagnostic pathway remains inconsistent. For instance, Brasme et al. identified pain as a factor that could lengthen the diagnostic pathway, suggesting that pain should be interpreted as a consequence of the tumor growth and size rather than a sole indicator [15]. In contrast, our studies align with studies by Pan et al. and Biscaglia et al., indicating that pain shortens the diagnostic timeline by prompting earlier consultation [17,18]. Sneppen et al. further refined this by distinguishing between constant and intermittent pain, showing that constant pain leads to shorter delays compared to intermittent pain [16]. The discrepancy in these findings underscores the complexity of symptom interpretation in sarcoma patients. The acceleration of diagnosis in patients experiencing pain may be attributed to pain being perceived as an alarming and disruptive symptom, prompting patients to seek medical attention promptly [6]. Pain is often associated with more aggressive or rapidly growing tumors, which may heighten patient concern [7,17]. In contrast, symptoms like swelling or sensory disturbances might be perceived as benign or attributed to less serious conditions, leading to delays in seeking care [7,19]. Swelling may be considered a normal response to injury or overuse, and sensory disturbances might be misattributed to neuropathies or musculoskeletal issues [6]. Our study also highlights that the impact of these symptoms varies between tumor types and locations. For example, growing swellings significantly delayed diagnosis in both BS and benign tumors of bone (BBT), suggesting that patients may not recognize the urgency of this symptom regardless of malignancy status. Additionally, integumentary symptoms led to substantial delays in patients with benign superficial tumors, indicating that skin changes are often underestimated in their potential seriousness. These findings have important clinical and public health implications. Improving patient awareness about the significance of symptoms like swelling and sensory disturbances is crucial. Educational campaigns and patient education materials should emphasize that persistent or unexplained symptoms warrant medical evaluation. For healthcare providers, particularly in primary and secondary care, enhancing the recognition of potential sarcoma symptoms is essential. Training programs and clinical guidelines may reinforce the importance of considering mesenchymal tumors in differential diagnoses when patients present with these symptoms. Optimizing diagnostic pathways involves developing clear referral protocols to ensure timely evaluation by specialists. Implementing standardized guidelines for imaging and referral can help reduce delays. For instance, any persistent pain, increasing swelling or restricted movement lasting more than four weeks should prompt consideration of BS and warrant further radiological investigation, as suggested by Hardes et al. [20]. The European Society for Medical Oncology (ESMO) clinical practice guidelines recommend appropriate imaging modalities—such as MRI for STS and conventional X-rays followed by MRI for BS—before invasive procedures like biopsies are performed [21,22]. From a public health perspective, our findings support the need for policy initiatives that promote early detection strategies. The centralization of care within a hub-and-spoke-model, as highlighted by studies showing improved outcomes when patients are managed at specialized sarcoma centers [23,24,25,26], should be encouraged. Healthcare policies should facilitate resource allocation to support these centers and streamline referral processes. A major strength of this study is the comprehensive analysis of a large, prospectively collected dataset from multiple institutions within the Swiss Sarcoma Network. By including both benign and malignant tumors and considering a wide range of symptoms and diagnostic intervals, we provide a nuanced understanding of the diagnostic pathway in mesenchymal tumors. The novel approach of grouping symptoms into clinically relevant categories adds depth to the analysis and highlights specific areas where interventions could be targeted. However, there are limitations to consider. The potential selection bias inherent in focusing on patients referred to a sarcoma center suggests that someone along the diagnostic pathway had already considered the possibility of a sarcoma, which may not reflect the experiences of all patients with mesenchymal tumors. Additionally, the granular nature of our analysis resulted in smaller sample sizes within certain tumor subgroups and symptom classes, which may have limited the statistical power to detect some associations. The retrospective design and reliance on medical records may also introduce biases related to symptom reporting and documentation. For example, symptoms were listed in relation to the tumor disease without screening for pre-existing conditions, such as peripheral neuropathy, that could explain symptoms like sensory disturbance. This unfiltered reporting approach may impact the specificity of symptom attribution to the tumor. The classification of “swelling” as a local increase in volume—excluding other common descriptors like “bulges, bumps and lumps”—may have led to deviations from the norm in tumor distribution and weakened comparability with other studies. Further research is warranted to delve deeper into how specific symptom characteristics influence diagnostic delays. For instance, distinguishing between types of pain (constant vs. intermittent) and their effects on PI and TI could provide more precise insights. Investigating whether these effects apply uniformly across all bone sarcomas or are limited to specific entities would enhance understanding. When considering tumor locations by body regions, the distribution in our study largely aligns with the literature. Minor differences include a slightly higher proportion of limb girdle tumors in BS and BSTT, as well as more deep trunk tumors in STS, while extremities are slightly less frequent. The occurrence of viscera tumors (2.8%) matches the rarity reported in the literature (<5%). These variations may reflect differences in patient population or classification criteria. Considering the broad range of tumor locations, from extremities to intraperitoneal regions and beyond, potential biases may have been introduced in assessing the effects of symptoms on diagnostic time intervals. Future studies could address this by examining the impact of symptoms on specific sarcoma subtypes and conducting a more detailed analysis of individual symptoms, such as “swelling”. Additionally, focusing on how the tumor location influences the perception of symptoms could yield valuable insights and contribute to the refinement of diagnostic pathways. Exploring patient-related factors, such as personality traits and health-seeking behaviors, could identify groups at a higher risk for diagnostic delays. Qualitative studies examining patient perceptions of symptoms and barriers to seeking care would be valuable. Additionally, research focusing on healthcare providers is crucial. Surveys and educational assessments could determine the level of awareness among general practitioners and specialists regarding mesenchymal tumors and associated symptoms. Investigating systemic barriers within the healthcare system that contribute to delays, such as access to imaging modalities or specialist referrals, would inform policy interventions. Longitudinal studies assessing the impact of diagnostic delays on long-term patient outcomes, including survival rates and quality of life, are also essential to fully understanding the implications of our findings.

## 5. Conclusions

In conclusion, this study demonstrates that pain accelerates the diagnostic process in mesenchymal tumors by prompting earlier medical consultation, while symptoms like swelling and sensory disturbances contribute to significant delays across various intervals of the diagnostic pathway. These findings highlight the need for increased awareness among both patients and healthcare providers regarding the potential seriousness of these symptoms. Implementing educational initiatives, optimizing referral protocols and promoting early detection strategies are crucial steps toward reducing diagnostic delays and improving patient outcomes. Addressing these challenges requires a concerted effort from clinicians, policymakers and public health professionals to enhance the care of patients with mesenchymal tumors.

## Figures and Tables

**Table 1:** Patient Characteristics by Tumor Groups.

	BS		STS		BBT		BSTT	
		Deep and Superficial STS	Deep STS	Superficial STS		Deep and Superficial BSTT	Deep BSTT	Superficial BSTT
	*n* = 82	*n* = 356	*n* = 296	*n* = 60	*n* = 61	*n* = 213	*n* = 172	*n* = 41
**Age, years**	44.0 (19.0, 65.0)	60.0 (46.0, 72.0)	60.0 (46.0, 72.0)	61.5 (42.3, 74.3)	34.0 (23.0, 45.0)	55.0 (44.0, 63.0)	56.0 (44.0, 65.0)	54.0 (44.0, 61.0)
**Female, (%)**	30 (36.6%)	178 (50.0%)	145 (49.0%)	33 (55.0%)	30 (49.2%)	110 (51.6%)	90 (52.3%)	20 (48.8%)
**Grade**								
G1	7 (8.5%)	67 (18.8%)	52 (17.6%)	15 (25.0%)	not applicable	not applicable	not applicable	not applicable
G2	5 (6.1%)	49 (13.7%)	41 (13.9%)	8 (13.3%)	not applicable	not applicable	not applicable	not applicable
G3	26 (31.7%)	100 (28.1%)	85 (28.7%)	15 (25.0%)	not applicable	not applicable	not applicable	not applicable
NA	44 (53.7%)	140 (39.3%)	118 (39.9%)	22 (36.7%)	not applicable	not applicable	not applicable	not applicable
**Region**								
Head and Neck	2 (2.4%)	19 (5.3%)	9 (3.0%)	10 (16.7%)	4 (6.6%)	11 (5.2%)	6 (3.5%)	5 (12.2%)
Superficial Trunk	-	22 (6.2%)	-	22 (36.7%)	-	6 (2.8%)	-	6 (14.6%)
Deep Trunk	15 (18.3%)	116 (32.6%)	116 (39.2%)	-	7 (11.5%)	34 (16.0%)	34 (19.8%)	-
Limb Girdle	32 (39.0%)	81 (22.8%)	73 (24.7%)	8 (13.3%)	18 (29.5%)	73 (34.3%)	59 (34.3%)	14 (34.2%)
Extremities	33 (40.2%)	108 (30.3%)	88 (29.7%)	20 (33.3%)	32 (52.5%)	89 (41.8%)	73 (42.4%)	16 (39.0%)
Viscera	-	10 (2.8%)	10 (3.4%)	-	-	-	-	-
**Size, mm**	60.0 (39.5, 85.0)	70.0 (32.0, 124.0)	86.0 (45.0, 130.0)	28.0 (20.0, 44.0)	31.5 (11.5, 50.5)	60.0 (38.3, 97.3)	61.0 (39.0, 100.5)	54.0 (35.5, 79.5)
0–50 mm, *n*	25 (30.5%)	106 (29.8%)	70 (23.6%)	36 (60.0%)	33 (54.1%)	83 (39.0%)	65 (37.4%)	18 (43.9%)
51–100 mm, *n*	30 (36.6%)	67 (18.8%)	59 (19.9%)	8 (13.3%)	10 (16.4%)	72 (33.8%)	55 (32.0%)	17 (41.5%)
101–150 mm, *n*	11 (13.4%)	50 (14.1%)	50 (16.9%)	0 (0.0%)	1 (1.6%)	29 (13.6%)	27 (15.7%)	2 (4.9%)
>150 mm, *n*	1 (1.2%)	42 (11.8%)	41 (13.9%)	1 (1.7%)	0 (0.0%)	14 (6.6%)	13 (7.6%)	1 (2.4%)
NA	15 (18.3%)	91 (25.6%)	76 (25.7%)	15 (25.0%)	17 (27.8%)	15 (7.0%)	12 (7.0%)	3 (7.3%)

Legend: NA—not applicable.

**Table 2:** More precise breakdown of symptoms per subgroup *.

	BS		STS		BBT		BSTT	
		Deep and Superficial STS	Deep STS	Superficial STS		Deep and Superficial BSTT	Deep BSTT	Superficial BSTT
	*n* = 82	*n* = 356	*n* = 296	*n* = 60	*n* = 61	*n* = 213	*n* = 172	*n* = 41
**circulatory**								
bleeding	0 (0.0%)	5 (1.4%)	2 (0.7%)	3 (5.0%)	0 (0.0%)	0 (0.0%)	0 (0.0%)	0 (0.0%)
hematoma	0 (0.0%)	4 (1.1%)	3 (1.0%)	1 (1.7%)	0 (0.0%)	0 (0.0%)	0 (0.0%)	0 (0.0%)
**gastrointestinal**								
constipation	1 (1.2%)	6 (1.7%)	6 (2.0%)	0 (0.0%)	0 (0.0%)	0 (0.0%)	0 (0.0%)	0 (0.0%)
increase in abdominal girth	0 (0.0%)	7 (2.0%)	7 (2.4%)	0 (0.0%)	0 (0.0%)	1 (0.5%)	1 (0.6%)	0 (0.0%)
loss of appetite	0 (0.0%)	5 (1.4%)	5 (1.7%)	0 (0.0%)	0 (0.0%)	0 (0.0%)	0 (0.0%)	0 (0.0%)
**general symptoms**								
difference in leg circumference	0 (0.0%)	7 (2.0%)	6 (2.0%)	1 (1.7%)	0 (0.0%)	2 (0.9%)	2 (1.2%)	0 (0.0%)
fatigue	0 (0.0%)	8 (2.3%)	8 (2.7%)	0 (0.0%)	0 (0.0%)	3 (1.4%)	3 (1.7%)	0 (0.0%)
fever	1 (1.2%)	4 (1.1%)	4 (1.4%)	0 (0.0%)	1 (1.6%)	0 (0.0%)	0 (0.0%)	0 (0.0%)
growing swelling	9 (11.0%)	88 (24.7%)	67 (22.6%)	21 (35.0%)	7 (11.5%)	80 (37.6%)	57 (33.1%)	23 (33.1%)
night sweats	2 (2.4%)	6 (1.7%)	6 (2.0%)	0 (0.0%)	0 (0.0%)	0 (0.0%)	0 (0.0%)	0 (0.0%)
pain	65 (79.3%)	138 (38.8%)	131 (44.3%)	7 (11.7%)	43 (70.5%)	78 (36.6%)	68 (39.5%)	10 (39.5%)
swelling	25 (30.5%)	210 (59.0%)	165 (55.7%)	45 (75.0%)	21 (34.4%)	160 (75.1%)	120 (69.8%)	40 (69.8%)
weight loss	0 (0.0%)	16 (4.5%)	16 (5.4%)	0 (0.0%)	0 (0.0%)	1 (0.5%)	1 (0.6%)	0 (0.0%)
**incidental finding**								
incidental finding	9 (11.0%)	46 (12.9%)	42 (14.2%)	4 (6.7%)	15 (24.6%)	32 (15.0%)	32 (18.6%)	0 (0.0%)
incidental finding after trauma	3 (3.7%)	2 (0.6%)	2 (0.7%)	0 (0.0%)	8 (13.1%)	3 (1.4%)	3 (1.7%)	0 (0.0%)
incidental finding at control of cancerous disease	2 (2.4%)	26 (7.3%)	23 (7.8%)	3 (5.0%)	3 (4.9%)	7 (3.3%)	7 (4.1%)	0 (0.0%)
**integumental**								
discoloration	1 (1.2%)	7 (2.0%)	0 (0.0%)	7 (11.7%)	0 (0.0%)	3 (1.4%)	2 (1.2%)	1 (2.4%)
exophytic tumor	0 (0.0%)	7 (2.0%)	3 (1.0%)	4 (6.7%)	0 (0.0%)	0 (0.0%)	0 (0.0%)	0 (0.0%)
pruritus	1 (1.2%)	2 (0.6%)	0 (0.0%)	2 (3.3%)	0 (0.0%)	1 (0.5%)	0 (0.0%)	1 (2.4%)
skin lesion	0 (0.0%)	7 (2.0%)	3 (1.0%)	4 (6.7%)	0 (0.0%)	0 (0.0%)	0 (0.0%)	0 (0.0%)
ulcerating swelling	0 (0.0%)	5 (1.4%)	0 (0.0%)	5 (8.%)	0 (0.0%)	0 (0.0%)	0 (0.0%)	0 (0.0%)
**musculoskeletal system**								
loss of function	13 (15.9%)	17 (4.8%)	17 (5.7%)	0 (0.0%)	12 (19.7%)	12 (5.6%)	10 (5.8%)	2 (4.9%)
loss of strength	4 (4.9%)	4 (1.1%)	4 (1.4%)	0 (0.0%)	1 (1.6%)	4 (1.9%)	4 (2.3%)	0 (0.0%)
**respiratory system**								
change of voice	1 (1.2%)	0 (0.0%)	0 (0.0%)	0 (0.0%)	0 (0.0%)	0 (0.0%)	0 (0.0%)	0 (0.0%)
dyspnea	0 (0.0%)	2 (0.6%)	1 (0.3%)	1 (1.7%)	0 (0.0%)	0 (0.0%)	0 (0.0%)	0 (0.0%)
**neurological**								
sensory disturbance	8 (9.8%)	40 (11.2%)	35 (11.8%)	5 (8.3%)	2 (3.3%)	42 (19.7%)	24 (14.0%)	18 (43.9%)
**urogenital**								
hematuria	1 (1.2%)	3 (0.8%)	3 (1.0%)	0 (0.0%)	0 (0.0%)	0 (0.0%)	0 (0.0%)	0 (0.0%)
pollakiuria	0 (0.0%)	4 (1.1%)	4 (1.4%)	0 (0.0%)	0 (0.0%)	0 (0.0%)	0 (0.0%)	0 (0.0%)
NA	3 (3.7%)	16 (4.5%)	15 (5.1%)	1 (1.7%)	1 (1.6%)	3 (1.4%)	3 (1.7%)	0 (0.0%)

* A shortened version of Table 2 is presented here. The complete version of Table 2, which was used for the statistical analysis, is available upon request.

**Table 3:** Influence of symptoms and symptom classes on length of intervals ’°.

								DI						TI	
		PI			PCI			SCI			TCI				
	Beta	95% CI	*p*-Value	Beta	95% CI	*p*-Value	Beta	95% CI	*p*-Value	Beta	95% CI	*p*-Value	Beta	95% CI	*p*-Value
**Bone sarcoma (*n* = 82)**															
Symptoms															
pain	−1787.5	−2812.4, −762.6	0.001	−226.4	−478.9, 26.1	0.08	−214.9	−457.9, 28.2	0.08	−8.0	−22.8, 6.8	0.29	−2556.8	−3788.5, −1325.2	<0.001
swelling	203.7	−628.0, 1035.4	0.63	176.0	−46.8, 398.8	0.12	174.7	−43.8, 393.1	0.12	−6.3	−19.5, 7.0	0.35	476.2	−579.1, 1531.6	0.37
growing swelling	1102.6	−74.7, 2280.0	0.07	433.2	124.1, 742.3	0.007	433.7	129.4, 737.9	0.006	2.5	−16.2, 21.2	0.79	1637.4	227.8, 3046.9	0.02
sensory disturbance	−300.7	1567.7, 966.2	0.64	549.5	209.6, 889.4	0.002	550.1	215.4, 884.8	0.002	−1.2	−22.5, 20.0	0.91	235.9	−1432.9, 1904.7	0.78
Symptomclass															
circulatory	−477.9	−3888.7, 2932.8	0.78	43.7	−877.6, 964.9	0.93	39.5	−869.1, 948.1	0.93	−1.5	−51.4, 48.5	0.95	−631.2	4541.1, 3278.6	0.75
gastrointestinal	−481.3	−2475.9, 1513.3	0.63	−3.0	−542.3, 536.2	0.99	−4.5	−536.2, 527.1	0.99	−7.2	−42.7, 28.4	0.69	−628.1	−3414.2, 2157.9	0.65
general	457.3	−1970.1, 2884.7	0.71	73.6	−268.3, 415.5	0.67	67.3	−252.5, 387.2	0.68	−13.7	−34.6, 7.3	0.2	600.7	−3309.5, 4510.9	0.76
integumental	90.99	−2338.7, 2520.7	0.94	−71.3	−727.0, 584.4	0.83	−75.0	−721.5, 571.6	0.82	−4.1	−39.7, 31.5	0.82	−174.5	−2965.1, 2616.0	0.9
musculoskeletal	−385.8	−1338.7, 567.0	0.42	−40.7	−304.7, 223.3	0.76	−37.2	−296.8, 222.4	0.78	19.1	3.36, 34.7	0.02	−271.1	−1613.5, 1071.3	0.69
respiratory	−478.9	−3889.7, 2931.8	0.78	−71.9	−993.0, 849.3	0.88	−31.4	−940.0, 877.1	0.95	−2.5	−52.5, 47.5	0.92	−705.5	−4614.4, 3203.5	0.72
neurological	−319.7	−1522.9, 883.5	0.6	478.0	154.3, 801.6	0.004	477.9	159.2, 796.6	0.004	−3.1	−22.9, 16.8	0.76	99.8	−1460.6, 1660.2	0.9
urogenital	268.3	−2160.7, 2697.2	0.83	−37.9	−693.8, 617.9	0.91	−36.5	−683.2, 610.2	0.91	6.8	−28.8, 42.3	0.71	68.1	−2722.8, 2858.9	0.96
**Soft tissue sarcoma (*n* = 356)**															
Symptoms															
pain	−258.6	−574.7, 57.4	0.11	−50.5	−155.5, 54.5	0.35	−80.3	−200.8, 40.2	0.19	33.0	−80.1, 146.1	0.57	−442.1	−853.4, −30.8	0.04
swelling	−15.0	−348.0, 317.9	0.93	108.1	4.5, 211.6	0.04	134.6	15.8, 253.4	0.03	28.8	−82.5, 140.0	0.61	52.2	−383.3, 487.7	0.81
growing swelling	118.4	−230.5, 467.2	0.51	62.1	−55.9, 180.1	0.3	127.3	−8.4, 263.0	0.07	138.4	12.8, 264.1	0.03	235.2	−220.2, 690.6	0.31
sensory disturbance	120.4	−347.5, 588.3	0.61	−82.4	−244.2, 79.4	0.32	−99.8	−283.2, 83.6	0.29	166.1	−8.3, 340.6	0.06	163.3	−460.7, 787.3	0.61
Symptomclass															
circulatory	594.7	−216.2, 1405.6	0.15	−80.8	−350.1, 188.6	0.56	−98.3	−411.2, 214.7	0.54	58.9	−220.5, 338.3	0.68	415.7	−571.8, 1403.3	0.41
gastrointestinal	−54.5	−679.0, 570.0	0.86	−78.3	−284.8, 128.3	0.46	−99.2	−339.1, 140.8	0.42	121.1	−112.4, 354.6	0.31	−24.0	−852.7, 804.8	0.96
general	209.2	−280.0, 698.5	0.4	72.7	−53.5, 199.0	0.26	88.2	−58.2, 234.5	0.24	91.6	−42.0, 225.2	0.18	252.8	0−379.9, 885.6	0.43
integumental	−27.2	−638.4, 584.0	0.93	98.5	−123.4, 320.5	0.38	97.1	−167.9, 362.0	0.47	53.4	−157.4, 264.1	0.62	78.7	−669.0, 826.3	0.84
musculoskeletal	83.6	−555.1, 722.4	0.8	−61.0	−277.5, 155.5	0.58	−79.3	−324.9, 166.2	0.53	168.8	−64.3, 401.9	0.16	258.2	−595.3, 1111.6	0.55
respiratory	−267.8	−1658.2, 1122.6	0.71	−78.0	−538.7, 382.8	0.74	−98.9	−634.4, 436.6	0.72	−229.4	−686.2, 227.3	0.32	−673.1	−2287.0, 940.9	0.41
neurological	82.3	−371.6, 536.2	0.72	−83.3	−241.2, 74.7	0.3	−100.2	−277.6, 77.3	0.27	163.4	−8.6, 335.4	0.06	140.8	−474.5, 756.2	0.65
urogenital	−328.3	−1141.1, 484.5	0.43	−79.8	−349.2, 189.5	0.56	−101.3	−414.3, 211.6	0.53	122.4	−169.8, 414.6	0.41	−321.1	−1355.3, 713.1	0.54
**Deep soft tissue sarcoma (*n* = 296)**															
Symptoms															
pain	−204.0	−544.8, 136.7	0.24	−50.5	−164.9, 64.0	0.39	−85.4	−218.5, 47.7	0.21	36.6	−95.0, 168.2	0.59	−426.2	−878.8, 26.4	0.07
swelling	−164.4	−518.9, 190.1	0.36	109.5	−4.5, 223.6	0.06	142.0	9.7, 274.4	0.04	39.4	−92.0, 170.8	0.56	−114.0	−591.5, 363.5	0.64
growing swelling	65.1	−325.3, 455.3	0.74	26.7	−109.2, 162.5	0.7	111.2	−47.2, 269.5	0.17	162.1	6.8, 317.4	0.04	170.2	−355.5, 695.9	0.52
sensory disturbance	−95.7	−592.6, 401.2	0.71	−81.4	−259.4, 96.6	0.37	−102.3	−306.5, 101.8	0.33	181.9	−17.3, 381.2	0.07	−24.8	−691.6, 642.0	0.94
Symptomclass															
circulatory	−1.4	−928.6, 925.8	0.99	−79.5	−396.0, 237.0	0.62	−99.5	−473.0, 273.9	0.6	63.3	−269.5, 396.1	0.71	−112.3	−1211.4, 986.8	0.84
gastrointestinal	6.1	−616.2, 628.4	0.99	−78.3	−290.3, 133.7	0.47	−100.9	−350.9, 149.1	0.43	125.7	−127.6, 378.9	0.33	39.0	−800.9, 878.9	0.93
general	110.9	−486.1, 707.9	0.72	65.1	−79.9, 210.1	0.38	83.8	−85.6, 253.1	0.33	139.1	−27.2, 305.4	0.1	138.4	−678.4, 955.2	0.74
integumental	−300.6	−1886.9, 1285.8	0.71	−43.0	−585.1, 499.1	0.88	−57.7	−697.7, 582.3	0.86	50.2	518.6, 619.0	0.86	−397.0	−2270.8, 1476.8	0.68
musculoskeletal	146.1	−490.0, 782.1	0.65	−60.8	−282.8, 161.3	0.59	−80.8	−336.5, 175.0	0.54	174.0	−78.8, 426.7	0.18	325.2	−538.7, 1189.1	0.46
respiratory	−167.7	−1754.3, 1419.0	0.84	−76.7	−618.8, 465.4	0.78	−98.8	−738.7, 541.1	0.76	−321.4	−888.6, 245.9	0.27	−680.9	−2553.1, 1191.3	0.47
neurological	−119.6	−599.7, 360.5	0.62	−82.4	−255.6, 90.8	0.35	−102.7	−299.3, 94.0	0.31	178.7	−17.5, 374.8	0.07	−41.8	−698.5, 614.8	0.9
urogenital	−271.9	−1079.1, 535.3	0.51	−79.5	−355.1, 196.1	0.57	−102.5	−427.7, 222.7	0.54	126.2	−189.9, 442.3	0.43	−263.0	−1307.9, 781.8	0.62
**Superficial soft tissue sarcoma (*n* = 60)**															
Symptoms															
pain	−230.3	−1429.3, 968.6	0.7	−87.8	−489.1, 313.5	0.66	−100.2	−507.8, 307.4	0.62	53.7	−176.1, 283.4	0.64	−215.0	−1630.1, 1200.1	0.76
swelling	743.1	−274.0, 1760.2	0.15	108.2	−164.4, 380.8	0.43	106.8	−178.6, 392.2	0.46	−35.1	−200.6, 130.5	0.67	804.1	−324.5, 1932.7	0.16
growing swelling	188.0	−634.0, 1010.0	0.65	204.2	−36.1, 444.4	0.09	206.2	39.2, 451.6	0.1	61.6	−92.1, 215.2	0.43	335.0	−627.6, 1297.5	0.49
sensory disturbance	1705.0	394.7, 3015.3	0.02	−87.8	−489.1, 313.5	0.66	−84.5	−492.4, 323.4	0.68	55.3	−259.8, 370.5	0.73	2037.6	199.2, 3876.1	0.03
Symptomclass															
circulatory	2324.5	682.96, 3966.10	0.006	−87.79	−595.32, 419.73	0.73	−94.42	−609.88, 421.04	0.71	39.66	−342.74, 422.056	0.84	2804.1	612.36, 4995.81	0.01
general	650.0	−274.7, 1574.7	0.16	108.2	−152.4, 368.7	0.41	108.7	−162.5, 379.9	0.42	−43.4	−202.4, 115.5	0.59	693.9	−368.2, 1756.0	0.2
integumental	−360.6	−1196.2, 475.0	0.39	163.5	−94.6, 421.7	0.21	175.5	−92.7, 443.7	0.2	57.3	−98.7, 213.3	0.46	−121.4	−1100.3, 857.5	0.8
respiratory	−665.7	−3655.0, 2323.6	0.66	−84.3	−946.3, 777.7	0.85	−98.4	−973.6, 776.8	0.82	44.0	−491.6, 579.5	0.87	−719.7	−3992.8, 2553.3	0.66
neurological	1705.0	394.7, 3015.3	0.01	−87.8	−489.1, 313.5	0.66	−84.5	−492.4, 323.4	0.68	55.3	−259.8, 370.5	0.73	2037.6	199.2, 3876.1	0.03
**Benign bone tumor (*n* = 61)**															
Symptoms															
pain	41.2	−270.3, 352.7	0.79	−83.9	−240.8, 72.9	0.29	−85.7	−242.0, 70.6	0.28	182.2	−375.7, 740.2	0.5	−308.5	−2010.2, 1393.2	0.71
swelling	241.1	−23.1, 505.4	0.07	77.4	−77.8, 232.5	0.32	73.6	−81.2, 228.4	0.34	−257.4	−777.0, 262.2	0.31	−341.8	−1913.9, 1230.3	0.65
growing swelling	383.0	17.8, 748.1	0.04	142.2	−76.06, 360.4	0.2	141.7	−75.9, 359.3	0.2	−143.4	−734.6, 447.7	0.61	301.1	−1401.0, 2003.3	0.71
sensory disturbance	108.4	−566.3, 783.1	0.75	−82.0	−475.5, 311.5	0.68	−85.7	−477.9, 306.6	0.66	−89.4	−937.5, 758.7	0.83	−10.5	−2429.0, 2408.1	0.99
Symptomclass															
gastrointestinal	−243.7	−1185.9, 698.6	0.61	−83.5	−635.0, 468.1	0.76	−87.1	637.0, 462.8	0.75	NA	NA	NA	NA	NA	NA
general	301.1	−249.3, 851.5	0.28	−24.3	−216.1, 167.6	0.8	−29.9	−221.1, 161.3	0.76	178.9	−982.6, 1340.5	0.75	NA	NA	NA
musculoskeletal	−120.1	−421.8, 181.5	0.43	−92.8	−270.4, 84.7	0.3	−95.9	−272.8, 81.0	0.28	−222.9	−807.1, 361.2	0.43	−915.8	−2550.0, 718.4	0.25
neurological	108.4	−566.3, 783.1	0.75	−82.0	−475.5, 311.5	0.68	−85.7	−477.9, 306.6	0.66	−89.4	−937.5, 758.7	0.83	−10.5	−2429.0, 2408.1	0.99
**Benign soft tissue tumor (*n* = 213)**															
Symptoms															
pain	−439.3	−962.4, 83.7	0.1	−11.0	−110.9, 89.0	0.83	−28.4	−131.3, 74.5	0.59	2.5	−40.7, 45.7	0.91	−388.7	−1094.6, 317.3	0.28
swelling	91.7	−663.4, 846.8	0.81	69.1	−43.4, 181.6	0.23	10.9	−103.7, 125.6	0.85	−25.2	−72.5, 22.0	0.29	203.4	−791.2, 1198.0	0.69
growing swelling	348.5	−176.0, 873.1	0.19	34.6	−67.6, 136.8	0.51	7.8	−97.8, 113.5	0.88	−34.6	−76.9, 7.7	0.11	377.3	−323.4, 1078.2	0.29
sensory disturbance	292.0	−323.8, 907.8	0.35	153.7	35.9, 271.5	0.01	134.3	10.3, 258.3	0.03	−41.0	−91.4, 9.5	0.11	584.0	−224.5, 1392.5	0.16
Symptomclass															
circulatory	−861.2	−4374.3, 2651.8	0.63	−69.1	−729.2, 591.0	0.84	−94.1	−788.5, 600.3	0.79	2.5	−254.9, 259.8	0.99	−1213.2	−5239.2, 2812.8	0.55
gastrointestinal	−870.3	−4383.3, 2642.7	0.63	−69.1	−729.2, 591.0	0.84	−61.9	−756.4, 632.6	0.86	NA	NA	NA	NA	NA	NA
general	NA	NA	NA	−24.3	−165.9, 117.3	0.74	−115.5	−254.1, 23.2	0.1	−44.6	−103.2, 14.0	0.14	NA	NA	NA
integumental	2447.1	898.8, 3995.5	0.002	−55.6	−438.8, 327.5	0.78	−73.8	−476.7, 329.2	0.72	30.7	−99.1, 160.6	0.64	2666.1	679.8, 4652.4	0.009
musculoskeletal	81.3	−863.8, 1026.4	0.87	114.6	−62.0, 291.2	0.2	98.5	−81.9, 278.8	0.28	13.0	−67.2, 93.2	0.75	562.0	−755.1, 1879.1	0.4
neurological	292.0	−323.8, 907.8	0.35	153.7	35.9, 271.5	0.01	134.3	10.3, 258.3	0.03	−41.0	−91.4, 9.5	0.11	584.0	−224.5, 1392.5	0.16
**Benign deep soft tissue tumor (*n* = 172)**															
Symptoms															
pain	−323.9	−878.3, 230.5	0.25	32.4	−46.9, 111.7	0.42	7.2	−81.6, 95.9	0.87	3.7	−48.7, 56.1	0.89	−208.8	−966.0, 548.4	0.59
swelling	−77.7	−816.0, 660.5	0.84	45.7	−40.6, 131.9	0.3	−12.3	−107.6, 83.0	0.8	−34.2	−88.9, 20.5	0.22	1.0	−971.2, 973.2	0.99
growing swelling	309.5	−259.8, 878.9	0.28	38.2	−45.5, 121.9	0.37	7.7	−86.5, 101.9	0.87	−58.7	−112.7, −4.7	0.03	383.3	−391.8, 1158.5	0.33
sensory disturbance	150.7	−587.2, 888.7	0.69	73.3	−35.9, 182.4	0.19	53.5	−70.9, 177.9	0.4	−88.6	−161.3, −16.0	0.02	399.6	−612.9, 1412.1	0.44
Symptomclass															
circulatory	−748.0	−4050.1, 2554.1	0.66	−49.9	−537.4, 437.6	0.84	−78.9	−634.5, 476.6	0.78	7.1	−279.0, 293.2	0.96	−1040.8	−4876.7, 2795.2	0.59
gastrointestinal	−757.1	−4059.1, 2544.9	0.66	−49.9	−537.4, 437.6	0.84	−46.8	−602.4, 508.9	0.87	NA	NA	NA	NA	NA	NA
general	NA	NA	NA	−47.9	−153.7, 58.0	0.37	−137.8	−249.3, −26.4	0.02	−52.1	−118.3, 14.2	0.12	NA	NA	NA
integumental	−370.3	−2290.9, 1550.4	0.7	−34.5	−380.3, 311.3	0.84	−61.7	−455.8, 332.3	0.76	5.6	−197.5, 208.7	0.96	−1061.9	−3783.6, 1659.9	0.44
musculoskeletal	89.9	−868.8, 1048.6	0.85	165.0	26.7, 303.4	0.02	144.1	−9.2, 297.3	0.07	15.6	−83.0, 114.2	0.76	771.7	−627.8, 2171.2	0.28
neurological	150.7	−587.2, 888.7	0.69	73.3	−35.9, 182.4	0.19	53.5	−70.9, 177.9	0.4	−88.6	−161.3, −16.0	0.02	399.6	−612.9, 1412.1	0.44
**Benign superficial soft tissue tumor (*n* = 41)**															
Symptoms															
pain	−579.5	−2123.0, 964.1	0.45	−225.5	−777.4, 326.5	0.41	−210.3	−724.4, 303.8	0.41	18.2	−12.7, 49.0	0.24	−466.4	−2612.5, 1679.6	0.66
swelling	1208.0	−3103.4, 5518.6	0.57	177.1	−1132.2, 1486.5	0.78	173.1	−1107.3, 1453.6	0.78	NA	NA	NA	NA	NA	NA
growing swelling	233.7	−1109.6, 1577.1	0.73	−47.2	−525.6, 431.3	0.84	−53.0	−512.9, 406.9	0.82	22.3	−1.5, 46.2	0.07	−60.0	−1784.0, 1663.9	0.94
sensory disturbance	213.6	−1130.1, 1557.3	0.75	290.6	−174.2, 755.5	0.21	287.9	−158.7, 734.6	0.2	7.8	−17.0, 32.6	0.52	376.7	−1319.7, 2073.0	0.65
Symptomclass															
integumental	6596.1	4350.1, 8842.2	<0.001	−165.8	−1475.3, 1143.8	0.8	−152.4	−1433.3, 1128.4	0.81	43.3	−4.8, 91.4	0.08	6216.6	3683.2, 8750.0	<0.001
musculoskeletal	399.9	−2697.3, 3497.0	0.8	−183.7	−1125.1, 757.7	0.69	−187.4	−1107.2, 732.5	0.68	3.7	−47.1, 54.5	0.88	−131.5	−3594.1, 3331.2	0.94
neurological	213.6	−1130.1, 1557.3	0.75	290.6	−174.2, 755.5	0.21	287.9	−158.7, 734.6	0.2	7.8	−17.0, 32.6	0.52	376.7	−1319.7, 2073.0	0.65

’ The influence of symptoms was analyzed across all sarcoma subgroups and intervals. Groups with insufficient symptom data to allow for statistically reliable analysis across all intervals (result NA) are not included in this table. ° For “symptoms”, “no pain/swelling/growing swelling” was used as a reference. For “symptomclass”, “no symptom of this class” was used as a reference.

## Data Availability

The data presented in this study are available on request from the corresponding author.

## Appendix A

Table A1 presents the median lengths of each interval within the diagnostic pathway, including the patient interval (PI), primary care interval (PCI), secondary care interval (SCI), tertiary care interval (TCI), diagnostic interval (DI) and total interval (TI) [10]. These baseline measurements provide context for the subsequent analysis of symptom impact on interval length.

**Table A1:** Length of the patient, diagnostic, primary care, secondary care, tertiary care and total interval in weeks.

	Overall	Bone Sarcoma		Soft-Tissue Sarcoma				Benign Bone Tumor		Benign Soft-Tissue Tumor			
				Deep and Superficial	Deep		Superficial			Deep and Superficial	Deep		Superficial
	*n* = 712	*n* = 82	*p*-Value ^a^	*n* = 356	*n* = 296	*p*-Value ^b^	*n* = 60	*n* = 61	*p*-Value ^c^	*n* = 213	*n* = 172	*p*-Value ^d^	*n* = 41
**Patient Interval, weeks**	90.0 (22.0, 284.0)	7.8 (2.7, 27.5)	0.46	8.8 (2.1, 29.0)	8.3 (2.0, 24.4)	**0.01**	20.7 (4.2, 130.6)	19.1 (4.3, 52.1)	0.17	21.6 (6.4, 109.6)	19.8 (6.3, 75.1)	0.22	29.9 (9.0, 176.4)
**Diagnostic Interval, weeks**	46.0 (25.5, 95.5)	7.6 (3.1, 14.2)	0.89	6.7 (3.7, 13.3)	6.9 (3.9, 13.7)	0.22	5.7 (3.6, 9.3)	19.8 (6.8, 79.7)	**0.005**	6.0 (3.6, 13.4)	6.0 (3.6, 14.6)	0.35	5.6 (3.6, 9.5)
**Primary** **Care** **Interval,** **weeks**	4.0 (0.0, 18.5)	0.6 (0.1, 6.5)	0.14	0.4 (0.0, 1.4)	0.4 (0.0, 1.3)	0.31	0.0 (0.0, 1.4)	0.8 (0.0, 44.9)	0.30	0.7 (0.0, 3.1)	0.7 (0.0, 4.4)	0.15	0.3 (0.0, 1.0)
**Secondary** **Care Interval,** **weeks**	26.0 (12.0, 57.0)	2.2 (0.9, 6.6)	**0.005**	4.3 (2.1, 9.1)	3.9 (1.9, 8.1)	**0.01**	8.1 (4.9, 10.2)	2.6 (1.0, 10.7)	0.47	3.5 (1.6, 7.5)	3.9 (1.7, 10.0)	0.14	2.6 (1.5, 3.8)
**Tertiary Care** **Interval,** **weeks**	14.0 (5.0, 26.3)	2.1 (1.0, 3.7)	**0.006**	1.3 (−0.6, 3.4)	1.6 (−0.2, 3.6)	**0.01**	0.9 (−3.3, 1.9)	3.1 (2.0, 8.1)	0.14	2.6 (1.8, 4.1)	2.6 (1.7, 4.0)	0.36	2.7 (1.9, 5.8)
Total Interval, weeks	213.0 (84.0, 762.2)	22.8 (11.9, 56.7)	0.82	23.3 (10.4, 59.4)	20.9 (10.4, 55.3)	0.07	34.8 (12.3, 148.0)	100.5 (48.1, 206.6)	0.22	48.2 (17.7, 193.3)	43.0 (14.7, 150.6)	**0.04**	138.1 (29.1, 304.4)

^a^: The *p*-value was calculated by a Wilcoxon rank sum test with continuity correction between STS (deep and superficial) and BS. ^b^: The *p*-value was calculated by a Wilcoxon rank sum test with continuity correction between deep STS and superficial STS. ^c^: The *p*-value was calculated by a Wilcoxon rank sum test with continuity correction between benign soft-tissue tumors (deep and superficial) and benign bone tumors. ^d^: The *p*-value was calculated by a Wilcoxon rank sum test with continuity correction between deep benign soft-tissue tumors and superficial benign soft-tissue tumors.

**Table A2:** Prevalence of Symptoms across Tumor Types.

Characteristic	BS		STS		BBT		BSTT	
		Deep and Superficial STS	Deep STS	Superficial STS		Deep and Superficial BSTT	Deep BSTT	Superficial BSTT
	*n* = 82	*n* = 356	*n* = 296	*n* = 60	*n* = 61	*n* = 213	*n* = 172	*n* = 41
**Symptom class**								
gastrointestinal	3 (3.7%)	21 (5.9%)	21 (7.1%)	0 (0.0%)	1 (1.6%)	1 (0.5%)	1 (0.6%)	0 (0.0%)
urogenital	2 (2.4%)	12 (3.4%)	12 (4.1%)	0 (0.0%)	0 (0.0%)	0 (0.0%)	0 (0.0%)	0 (0.0%)
circulatory	1 (1.2%)	12 (3.4%)	9 (3.0%)	3 (5.0%)	0 (0.0%)	1 (0.5%)	1 (0.6%)	0 (0.0%)
general	72 (87.8%)	279 (78.4%)	237 (80.1%)	42 (70.0%)	46 (75.4%)	182 (85.5%)	141 (82.0%)	41 (100.0%)
integumental	2 (2.4%)	22 (6.2%)	3 (1.0%)	19 (31.7%)	0 (0.0%)	5 (2.4%)	3 (1.7%)	2 (4.9%)
neurological	9 (11.0%)	43 (12.1%)	38 (12.8%)	5 (8.3%)	2 (3.3%)	42 (19.7%)	24 (14.0%)	18 (43.9%)
respiratory	1 (1.2%)	4 (1.1%)	3 (1.0%)	1 (1.7%)	0 (0.0%)	0 (0.0%)	0 (0.0%)	0 (0.0%)
musculoskeletal	17 (20.7%)	0 (0.0%)	0 (0.0%)	0 (0.0%)	0 (0.0%)	0 (0.0%)	0 (0.0%)	0 (0.0%)
incidental finding	9 (11.0%)	47 (13.2%)	43 (14.5%)	4 (6.7%)	15 (24.6%)	32 (15.0%)	32 (18.6%)	0 (0.0%)
NA	0 (0.0%)	4 (1.1%)	3 (1.0%)	1 (1.7%)	0 (0.0%)	0 (0.0%)	0 (0.0%)	0 (0.0%)
